# Distinguishing allergic dermatitis from atypical mycobacterial infection in tattoo reactions

**DOI:** 10.1016/j.jdcr.2025.07.020

**Published:** 2025-09-22

**Authors:** Makenna C. Chapman, Bobak Hedayati, Mary Horner, Suzanne Kilmer

**Affiliations:** a University of California Irvine School of Medicine, Irvine, California; b Davis Department of Dermatology, University of California, Davis, California; c Dermatology Consultants of Sacramento, Sacramento, California

**Keywords:** allergic reaction, laser, mycobacterium, tattoo

## Introduction

Tattoos have served as a cultural and self-identification symbol for over 5000 years and are now even utilized in medical applications, such as radiation treatment markers.^1^ Despite their widespread popularity, tattoos carry inherent risks. Allergic reactions to tattoo inks are well-documented in the medical literature and are frequently managed with ablative laser therapy.^2^ Another potential complication is nontuberculous mycobacterial (NTM) infection, which can closely mimic an allergic reaction to tattoo ink. Here, we report 2 cases of tattoo-related mycobacterial infections mimicking tattoo ink allergy resulting from ink contamination.

## Case report

**Fig 1:**
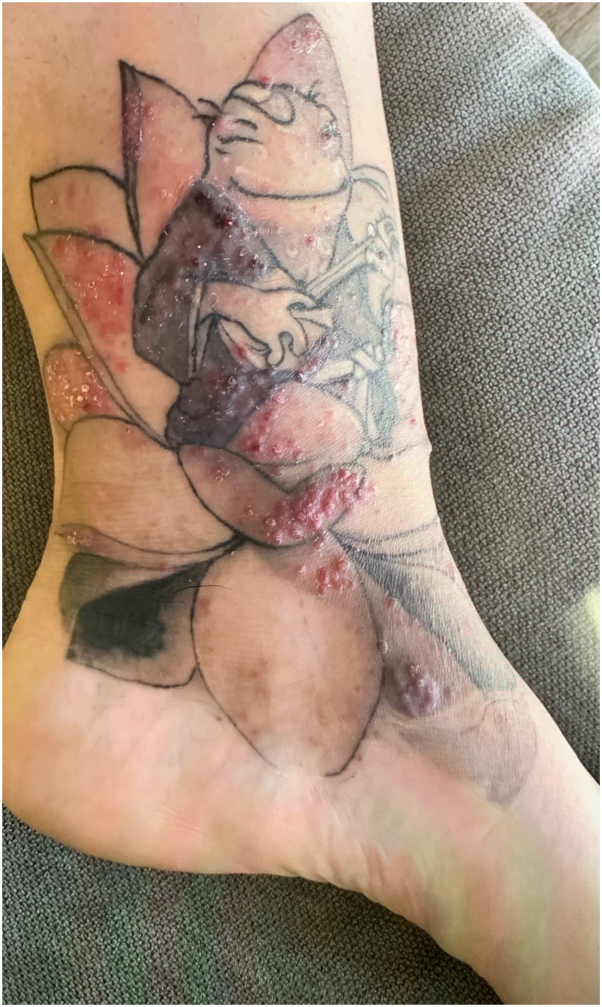
Initial presentation of Case 1. Multiple discrete erythematous papules coalescing into plaques confined to the *white ink* portions of a left ankle tattoo.

**Fig 2:**
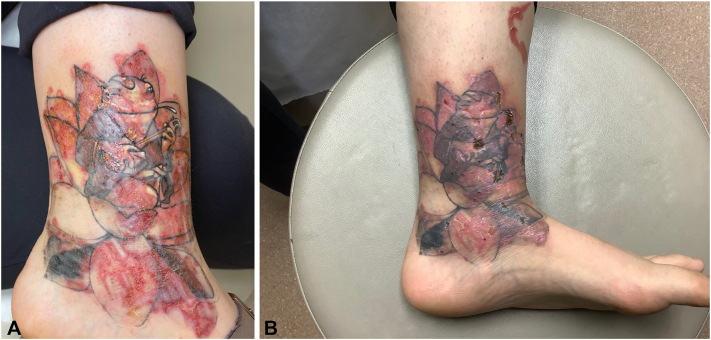
**A,** Six days after multipass fully ablative erbium laser treatment in Case 1. The treated area demonstrates erythema, crusting, and early re-epithelialization. **B,** Two weeks post-treatment in Case 1. There is continued re-epithelialization with mild residual erythema.

**Fig 3:**
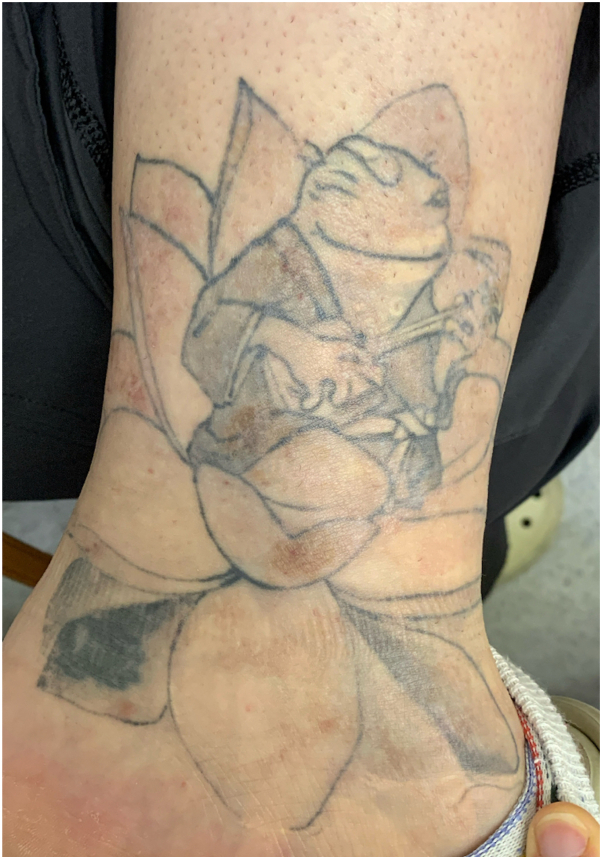
Recurrence in Case 1. Dull erythematous to *brown plaques* and nodules are present in the previously treated *white ink* regions of the tattoo.

**Fig 4:**
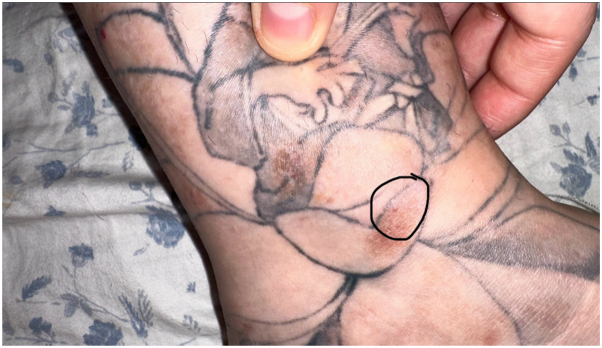
Closer view of the recurrence in Case 1. Dull erythematous to *brown* indurated plaques and nodules are localized to the *white ink* areas.

Case 1: A 29-year-old female with a history of atopic dermatitis and psoriasis requiring frequent potent topical steroid use, and ankylosing spondylitis requiring methotrexate and remicade, presented to the clinic 1 month after being tattooed on her left ankle with the formation of “very itchy red bumps” only in the areas of white ink used for shading and no systemic symptoms (Fig 1). It was initially diagnosed as an allergic tattoo ink reaction, and she was referred to a specialty laser clinic for a laser treatment to remove the allergic ink. Prior to treatment, the pathology was reviewed and was consistent with a tattoo ink allergic reaction including spongiotic dermatitis with scattered eosinophils. She received fully ablative erbium laser to remove ink in the areas of the suspected tattoo ink allergy (Fig 2, *A* and *B*). Immediately post multipass fully ablative erbium laser treatment she received mupirocin 2% topical ointment twice daily and cephalexin 500 mg capsule by mouth twice daily. One week post-treatment, she returned to the clinic with increasing pain and itching accompanied by a yellow purulent discharge with erythema in the treated area. Due to her signs of infection, she was switched to gentamicin 0.1% topical ointment on the skin twice daily, and doxycycline hyclate 100 mg capsule by mouth twice daily. Within the same week, the referring physician became aware of a local tattoo parlor having contaminated ink with mycobacterium chelonae, because the referring physician had a second patient (case 2) present with a similar reaction. The patient remained asymptomatic for a little over a year after the initial laser treatment, but then she developed a mild recurrence of her rash (Figs 3 and 4). Given the new knowledge of NTM ink contamination, the referring physician performed a tissue culture which showed presence of mycobacterium chelonae growth. She was treated with 500 mg oral azithromycin daily and 500 mg oral levofloxacin 500 mg daily for 3 months to a full recovery.

**Fig 5:**
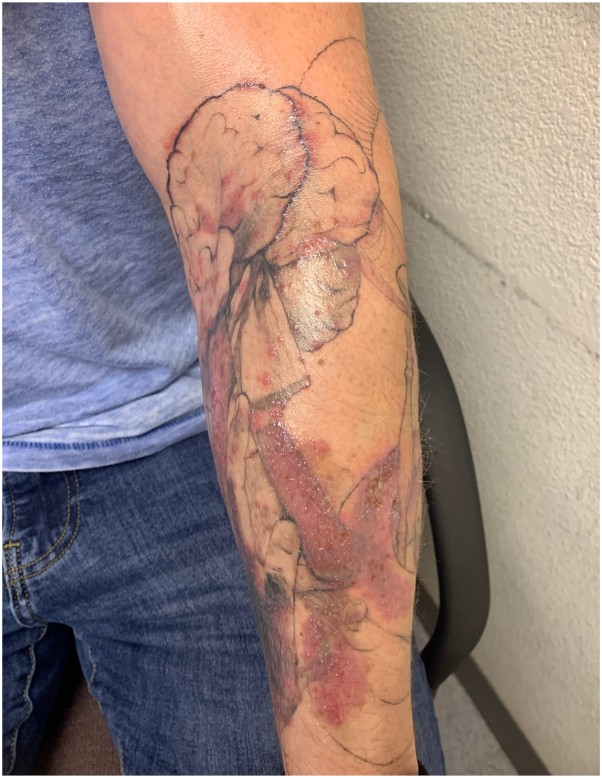
Initial presentation of Case 2. Well demarcated clusters of *pink to red papules* and plaques on the left forearm overlying *white ink*.

Case 2: A 30-year-old male presented to the clinic with a 1-month history of pink to red clustered papules and 1 pink nodule over a new tattoo on his left forearm from white ink used for shading. He presented with only cutaneous symptoms, none that were systemic. The reaction started 2 weeks after obtaining the tattoo. The reaction was described as very pruritic and with a burning sensation (Fig 5). He started oral prednisone and triamcinolone cream. With no improvement, he was then started on Keflex, but the reaction continued to progress. A biopsy came back as mixed, acute, chronic and granulomatous inflammatory infiltrate with exogenous tattoo pigment. Early culture results showed positive acid-fast bacillus (AFB), which later grew out to be confirmed as mycobacterium chelonae. The patient was then started on linezolid 600 mg tablets, azithromycin 500 mg tablets, and Vitamin B-6 50 mg tablets, on which he fully recovered. The tattoo parlor was contacted, and they confirmed several other probable infections reported from the same white ink color.

## Discussion

Tattoo related complications have been increasingly reported in the medical literature, yet distinguishing between an allergic reaction to tattoo ink and a mycobacterial infection from contaminated ink remains a clinical challenge.^3^^,^^4^ Both conditions can present with erythematous, pruritic, and inflamed skin changes, opening the possibility for a misdiagnosis and delayed appropriate treatment. Given the growing popularity of tattoos and the lack of standardized regulation surrounding tattoo ink and sanitation practices, it is crucial for clinicians to maintain a high index of suspicion when evaluating tattoo-associated skin reactions.

When patients present with a pruritic erythematous presentation around a newly administered tattoo, it is common for the first differential to be an allergic reaction to the ink in the tattoo. It is important to take a clinical history to differentiate between an inflammatory process from an exposure to infectious agent which takes a few weeks to a month, and an allergic process which is considered a ‘late’ reaction.^5^ Allergic reactions to tattoo ink often present as plaque-like, hyperkeratotic, or ulceronecrotic lesions, and may exhibit eczematous or lichenoid features [3]. While red ink is most frequently implicated, these reactions are not exclusive to that color.^5^ Allergic reactions to tattoo ink can be treated surgically through dermatomal shaving or with fully ablative resurfacing lasers to remove the offending ink.^6^^,^^7^ Ablative lasers can be used to remove the tattoo because it vaporizes the tissue and does not cause the ink to enter circulation, unlike Q-switched or pico-second lasers, the gold standards for scarless tattoo removal. These lasers with their ultrashort pulse durations shatter the ink, releasing it into the bloodstream and lymphatics which can then cause a systemic allergic reaction with full body eczematous or urticarial eruption to even anaphylaxis.^6^^,^^7^ Systemic reactions have also been noted with fractional ablative laser treatment which is typically used to penetrate deeper than a fully ablative laser.^8^^,^^9^

Patients infected with NTM can have a wide variety of presentations and laboratory results, further impressing the need for a thorough work up.^10^ Patients with an NTM infection may present with erythema, edema, pustules, scabs, small papules, or edematous and indurated plaques.^10^ Patients exposed to NTM contaminated ink or experiencing an ink allergy will develop inflammation localized to a single ink type-either due to contamination or an allergenic ingredient within that specific ink. A punch biopsy and a tissue culture for AFB must be done to confirm diagnosis of the NTM infection.^10^ While granulomatous inflammation is a classic histopathologic feature of NTM infections, its absence does not exclude the diagnosis. A significant study of 28 cutaneous NTM cases found that a marked granulomatous inflammatory reaction occurred in only 83% of immunocompetent and 60% of immunosuppressed patients, meaning up to 40% of cases may lack typical granulomas. Our first patient is immunocompromised, decreasing her odds of showing a granulomatous inflammatory reaction. The absence of granulomas clouded the initial diagnosis, as the histologic pattern overlapped with what is seen in tattoo pigment reactions (mixed inflammatory infiltrate, dermal changes). This underscores the critical need for microbiologic testing when clinical suspicion for infection remains despite nonspecific histopathology.^11^

Treatment of *Mycobacterium chelonae* soft tissue infections typically involve a combination of oral antibiotics, such as macrolides (clarithromycin or azithromycin), often with doxycycline, linezolid, or a fluoroquinolone, based on susceptibility testing. In more severe cases, an initial course of intravenous agents such as amikacin, imipenem, or tigecycline may be required. Total treatment duration generally ranges from 4 to 6 months, with 2 to 6 weeks of parenteral therapy followed by an extended course of oral antibiotics, adjusted according to clinical response.^12^^,^^13^

One of the underlying issues contributing to tattoo-related adverse reactions is the lack of regulatory oversight in the tattoo industry. Tattoo inks are classified as cosmetics by the Federal Drug Administration, not as medical, which allows for looser regulations and reliance on public reporting for adverse events.^14^ This lack of tight regulation allows tattoo ink to be full of polycyclic aromatic hydrocarbons, heavy metals, primary aromatic amines and even some unknown ingredients.^15^ Primary aromatic amines are reported to be carcinogenic agents and heavy metals are associated with neurodegenerative diseases, cancer, cardiovascular issues, and more.^15^ There is also a lack of supervision and law enforcement around the practice of tattoo art, allowing misconduct like not sanitizing needles, or diluting ink with tap water to remain an issue. This broad lack of regulation allows for substances in the ink that may be causing allergic reactions, as well as possible contamination.

## Conclusion

Allergic reactions and infections can both present with erythematous and pruritic lesions, but infections such as *Mycobacterium chelonae* may also involve nodules, pustules, systemic symptoms like fever, and typically appear within a few weeks of tattooing. Allergic reactions, on the other hand, may present later and persist chronically. However, these distinctions are not always reliable, and tissue biopsy with culture is essential for accurate diagnosis. It is imperative to perform tissue cultures on suspicious tattoo-related skin reactions before proceeding with laser or surgical tattoo removal. Patients experiencing a delayed or persistent inflammatory reaction should undergo biopsy and microbiologic evaluation to rule out infection. Misdiagnosing an NTM infection as an allergic reaction may result in unnecessary ablative laser tattoo removal, which is painful, expensive, can possibly lead to scarring, and most importantly, potentially worsen disease. Early antibiotic treatment could effectively resolve the infection without the need for surgical intervention. Clinicians should maintain a high degree of suspicion, especially when tattoo-associated lesions fail to respond to conventional treatments, ensuring that patients receive timely and appropriate therapy.

## Conflicts of interest

None disclosed.
